# Characterization and Valorization of the Microalgal Co-Product Spirugrass^®^: Protein Profile, Iron Speciation, and Potential Use as a Supplement for Honeybees

**DOI:** 10.3390/md23110443

**Published:** 2025-11-18

**Authors:** Thomas Dalmonte, Cecilia Rudelli, Daniele Alberoni, Angelica Lembo, Imma Gifuni, Giulia Andreani, Massimo Castellari, Gloria Isani

**Affiliations:** 1 Department of Veterinary Medical Sciences, Alma Mater Studiorum-University of Bologna, Via Tolara di Sopra 50, Ozzano dell’Emilia, 40064 Bologna, Italy; 2 Department of Agricultural and Food Sciences, Alma Mater Studiorum-University of Bologna, Viale Fanin 42, 40127 Bologna, Italy; daniele.alberoni@unibo.it; 3 Algosource Technologies, Le Frostidié, 44410 Assérac, France; 4 Interdepartmental Centre for Agri-Food Industrial Research, University of Bologna, Bologna, Via Quinto Bucci 336, 47521 Cesena, Italy; 5 IRTA–Food Safety and Functionality, Finca Camps i Armet, 17121 Girona, Catalonia, Spain; massimo.castellari@irta.cat

**Keywords:** *Limnospira platensis*, circular economy, protein characterization, honeybee nutrition and health, feed supplementation

## Abstract

Microalgae are used as dietary supplements for humans and animals, due to their excellent nutritional profile. This research aims to characterize Spirugrass^®^, a co-product obtained after the extraction of phycocyanin from *Limnospira platensis*, and to evaluate whether a stabilization treatment involving high-pressure processing (HPP) affects its proteomic profile. The research also aims to evaluate the possible use of Spirugrass^®^ as feed integration for honeybee health. Proteins were extracted and fractionated using size exclusion chromatography (SEC) and sodium dodecyl sulfate polyacrylamide gel electrophoresis (SDS-PAGE). The iron content was measured using atomic absorption spectrometry (AAS). Samples of Spirugrass^®^ were subjected to HPP at 600 MPa for five minutes and the effect on the integrity of the protein profile was analyzed. Finally, nine groups of 30 newly emerged honeybees were supplemented with Spirugrass^®^ in a controlled laboratory experiment. SDS-PAGE and SEC enabled the assessment of the quality and integrity of the Spirugrass^®^ proteome, which contains 80% of the proteins found in the algal biomass, including phycocyanin. The phycocyanin purity indices were 2.07 ± 0.14 and 0.72 ± 0.13 for the *L. platensis* and Spirugrass^®^ extracts, respectively. Spirugrass^®^ maintains a consistent iron content of 261 ± 15 μg/g dry weight, equivalent to 74% of the iron present in the algal biomass. In both *L. platensis* and Spirugrass^®^, iron was predominantly bound to high-molecular-mass proteins, including phycocyanin. Following HPP treatment, differences in the protein profiles were observed, which suggests partial protein degradation. Preliminary data obtained with honeybees are encouraging, as no mortality or adverse effects were observed and Spirugrass^®^ can be considered a promising candidate as feed supplement. Overall, the presence of consistent levels of soluble proteins, as well as protein-bound iron, suggests that Spirugrass^®^ could be used in animal nutrition.

## 1. Introduction

Microalgae are known and appreciated as dietary supplements due to their nutritional profile, which includes a high content of proteins, ω-3 fatty acids, macro (Ca, Mg, P) and trace (Fe, Zn, Cu, Se) elements, and other essential biomolecules, in particular, vitamins D and E [1,2,3,4]. In addition, microalgae may represent a source of still-unexplored biomolecules with unique properties, suggesting interesting applications in the food sector. Currently, microalgae are also being considered as alternative feed supplement for animals [5], including honeybees [6], and have been already added to animal feeds for dogs, cats, poultry, pigs, rabbits, and fish [7,8].

Novel sources of proteins are needed as the increasing demand for proteins raises the awareness of the risk of protein shortages. Some microalgae, e.g., *Limnospira platensis* or *Chlorella vulgaris*, have a protein percentage higher than 60% of the biomass [5] and can be considered as a promising alternative. However, the production costs of microalgae are still too high to be competitive with other sources, such as soy. Therefore, the reuse of microalgal co-products derived from the extraction of high-value biomolecules, such as phycocyanin or carotenoids, may be attractive to reduce the costs and promote circular economy.

*Limnospira platensis* is a filamentous, photosynthetic cyanobacterium and the most widely cultivated microalga for commercial use, valued for its exceptional nutritional composition rich in high-quality proteins, essential amino acids, pigments such as phycocyanin, vitamins, minerals, and bioactive compounds [9]. Spirugrass^®^, a co-product remaining after phycocyanin extraction from *L. platensis* biomass, can be considered as an interesting ingredient for feed integration. Compared to intact algal biomass, Spirugrass^®^ has the advantage that the cell structure has already been partially disaggregated, thus destroying the cell membrane and improving the digestibility of the product. In addition, the Spirugrass^®^ provided by Algosource comes out from a solvent-free extraction process that preserves the structure and functionality of the molecules. However, a stabilization step is required to reduce the total microbial load, improve the stability of this co-product over time, and make it compliant with the food and feed specification requirements [10]. High-pressure processing (HPP), a method using hydrostatic pressures between 100 and 800 MPa for short durations [11], can be considered as an interesting option to reduce the microbial load while maintaining the nutritional characteristics of the product.

The nutritional potential of this co-product is still unexplored and, to validate this potential, it is important to assess the quality of its proteins and the effect of its supplementation. In this study, the target species identified for in vivo testing of Spirugrass^®^ as feed supplement is *Apis mellifera*, the domestic honeybee, which has suffered population declines in recent years due to a number of factors. Among the most important are parasites and pathogens, which are influenced by beekeeping management practices. In addition, land use around colonies [12], together with urbanization and intensive agricultural practices [13,14], affects forage quality and increases pesticide exposure. Finally, climate change, along with agricultural expansion and the introduction of invasive species [15], is considered a major driver of biodiversity loss because it affects species distribution and abundance [16]. In this complex scenario, honeybee colonies are facing shortage of nectar and pollen more frequently than in the past, and supplementation is required to support the colonies. Indeed, a balanced diet is necessary for the health and the wellbeing of honeybees, which face negative effects, such as metabolic imbalances [17] and immune deficiencies [18]. It is therefore of capital importance to find new, effective supplements to support honeybees, especially when they are under stress.

The working hypothesis was that Spirugrass^®^, a co-product derived from *L. platensis* biomass processing, is a source of protein and bioavailable iron with potential benefits for animal nutrition. However, several knowledge gaps remain regarding (i) the impact of HPP on Spirugrass^®^ protein aggregation and hydrolysis; (ii) the nutritional relevance of iron associated with high-molecular-mass proteins such as phycocyanin; and (iii) the measurable outcomes in honeybees that reflect improved health status. Therefore, this study aims to (i) characterize Spirugrass^®^ with emphasis on its soluble protein profile and iron speciation compared with whole algal biomass; (ii) assess the influence of HPP treatment on its proteins; and (iii) evaluate its potential as a feed supplement to support honeybee health.

## 2. Results and Discussion

### 2.1. Characterization of Spirugrass^®^ and Comparison with Algal Biomass

Microalgae have complex cell walls, which require to be disrupted, either mechanically or non-mechanically, to release a mixture of soluble molecules, including proteins, from the cells into the surrounding medium [19]. In contrast to the process commonly used for the extraction of these molecules from *L. platensis*, which is based on disruption procedures such as sonication or ultrasonication [9,20], a milder extraction was chosen for Spirugrass^®^, as it is a residue of the extraction of phycocyanin from the algal biomass. The total soluble protein content was found to be 415 ± 31.7 mg/g dw in the algal biomass and 333 ± 21.3 mg/g dw in Spirugrass^®^. The lower content of soluble proteins in Spirugrass^®^ is due to it being the downstream co-product of the phycocyanin extraction process used to produce Spirulysat^®^, which had a protein concentration of 11.1 ± 0.46 mg/mL.

#### 2.1.1. SDS-PAGE of Soluble Extracts

**Figure 1:**
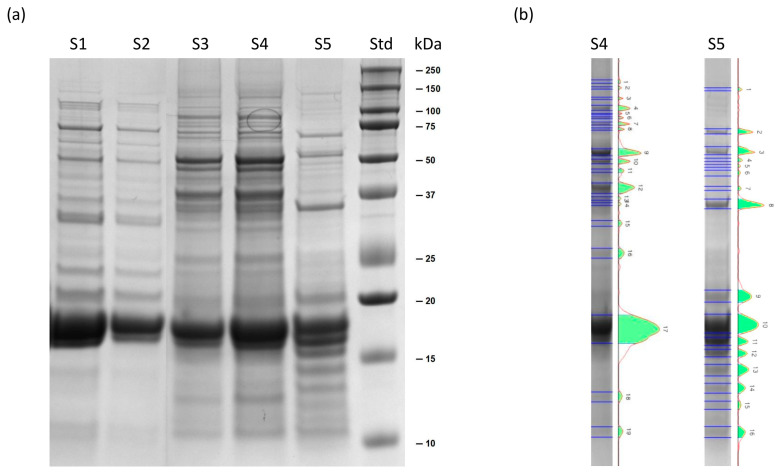
Representative SDS-PAGE gel (4–12%, Comassie staining): (**a**) from left to right, Spirulysat^®^ diluted 1:5 and 1:10 (S1, S2), two *L. platensis* extracts diluted 1:10 (S3) and 1:5 (S4) and one Spirugrass^®^ extract diluted 1:5 (S5); the molecular mass marker is reported in lane 6 (Std); (**b**) pherograms of S4 (*L. platensis* extract diluted 1:5) and S5 (Spirugrass^®^ extract diluted 1:5) are reported as examples to highlight the differences between the two matrices.

SDS-PAGE was performed on the soluble proteins extracted from *L. platensis* and Spirugrass^®^ to check the quality and the integrity of the proteome. As suggested in a previous paper by the authors [9], the electrophoretic profile can serve as an interesting molecular fingerprint to evaluate the quality and integrity of the proteome of an extract and can be used to compare samples obtained from a sequential process. The quality of the extracted proteins can be appreciated in Figure 1, which shows an SDS-PAGE gel and selected pherograms of extracts from *L. platensis* (S3 and S4) and Spirugrass^®^ (S5). For comparison, the profile of Spirulysat^®^, the product obtained after the extraction of phycocyanin and other biomolecules from *L. platensis*, is also shown in the gel. In all the samples, including Spirugrass^®^, the most abundant bands are at 18 and 16 kDa. These bands contain phycocyanin as reported by Isani et al. (2022) [21]. The protein profile of *L. platensis* extracts is similar to that reported by Aiello et al. (2019) [22], who found two intense bands corresponding to α and β subunits of phycocyanin, at 18 and 16 kDa, and most of the other protein bands in the range of 55–35 kDa. Moreover, Mohammadi et al. (2022) [23] reported that the profile of the unhydrolyzed protein extract of *L. platensis* showed several high intensity bands at 85, 40, and 25 kDa, while the electrophoretic pattern of extracts obtained by Htoo et al. (2024) [20], produced using different extraction procedures, showed bands at molecular mass of 75 kDa, 50 kDa, 37 kDa, 25 kDa, 20 kDa, and 15 kDa.

No intense protein degradation was detected in Spirugrass^®^ extracts. However, the electrophoretic profile shows a lower phycocyanin peak, fewer bands in the 55–35 kDa range, and a higher number of bands of lower molecular mass. These features suggest partial fragmentation of high-molecular-mass proteins or, alternatively, disaggregation of complex molecular structures, likely caused by the extraction process used to obtain Spirulysat*^®^*, whose profile is mainly characterized by bands with molecular masses higher than those of phycocyanin (Figure 1a).

**Figure 2:**
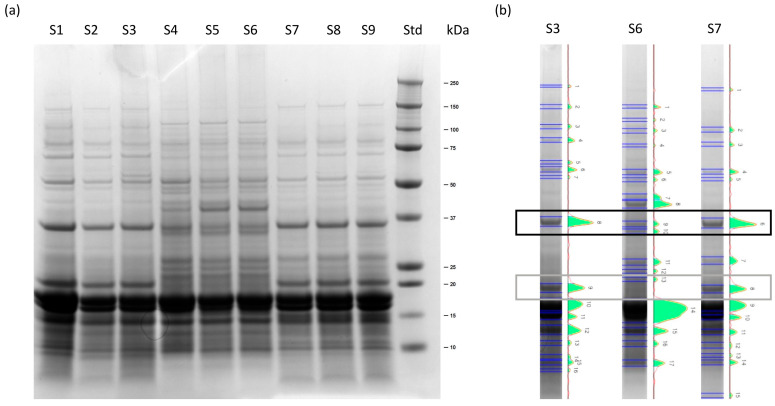
Representative SDS-PAGE gel (4–12%, Comassie staining): (**a**) extracts of Spirugrass^®^ from three different batches, B2 (S1, S2, S3), B4 (S4, S5, S6), and B1 (S7, S8, S9); standard of molecular mass (Std). Each batch was extracted and analyzed in triplicate. (**b**) Pherograms of S3, S6, and S7 are reported as examples highlighting the differences among the batches. The black rectangle indicates the 35 kDa band, while the gray rectangle indicates the 20 kDa band.

To assess the protein variability in three batches of Spirugrass^®^, the extracts were fractionated using SDS-PAGE (Figure 2). The profiles obtained were indicative of good quality, without massive protein degradation. Bands of 18 and 16 kDa are present in all the samples, due to the presence of phycocyanin α and β-subunits. Differences in high- and medium-molecular-mass protein bands were observed in extracts from batch B4; in particular, the band at 20 kDa is lacking and that at 35 kDa is less intense. Differences in SDS-PAGE profiles of extracts obtained from different commercial samples of *L. platensis* have also been previously reported by Isani et al. (2022) [9], suggesting that what we have found in different Spirugrass^®^ samples may be due to differences in the starting biomasses. Anyway, all the batches were characterized by the presence of phycocyanin and did not show protein degradation.

#### 2.1.2. Size Exclusion Chromatography

**Figure 3:**
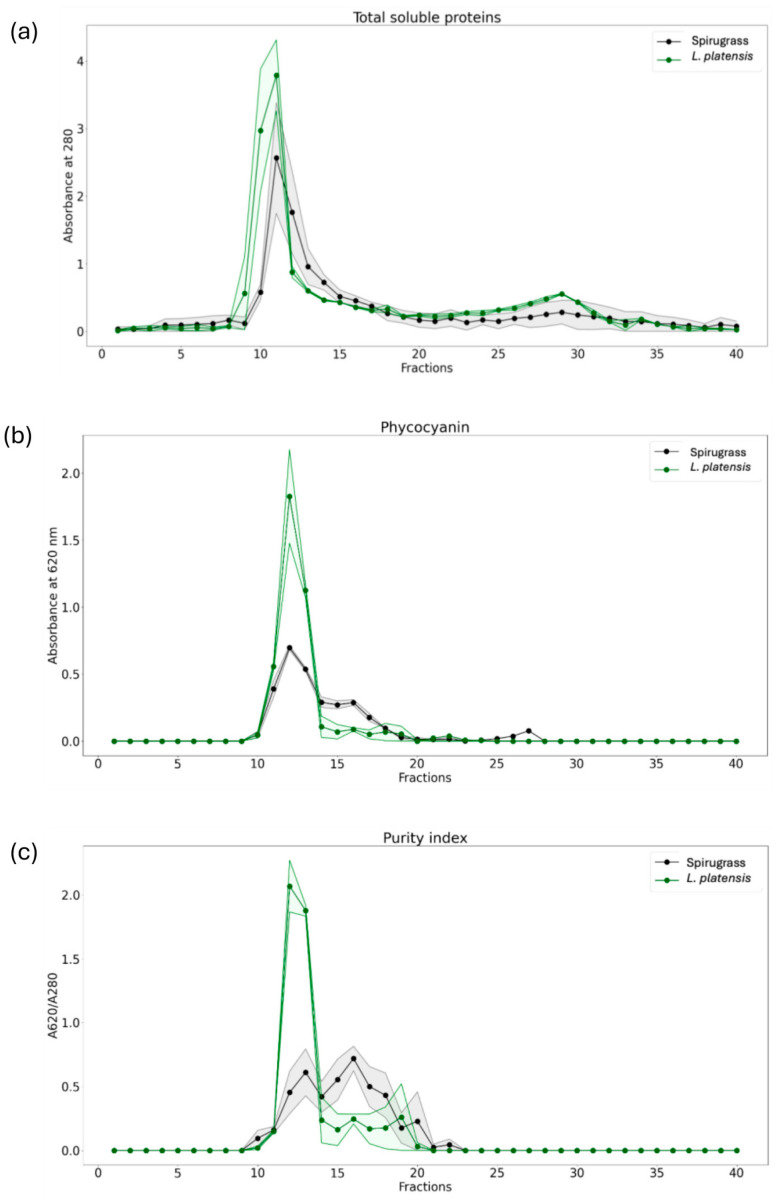
(**a**) Chromatographic patterns of proteins detected at 280, (**b**) phycocyanin detected at 620 nm, and (**c**) the purity index in fractions of SEC of extracts obtained from Spirugrass^®^ and *L. platensis* biomass. The shaded area surrounding the chromatograms indicates the variability between replicates.

Unlike SDS-PAGE, which denatures proteins, non-denaturing size exclusion chromatography (SEC) allows soluble proteins to be separated in their native state. The profile of proteins determined by reading the absorbance at 280 nm was similar between Spirugrass^®^ and *L. platensis* extracts (Figure 3a). The presence of phycocyanin in Spirugrass^®^ was confirmed after the analysis of the fractions at 620 nm of absorbance. Phycocyanin was present between fractions 10 and 14 which contain proteins with molecular masses between >75 and 60 kDa, in agreement with previous findings [21] (Figure 3b). In fact, under the non-denaturing conditions of SEC, phycocyanin appears in the form of polymers of high molecular mass. The peak of phycocyanin in Spirugrass^®^ was lower as compared to that contained in the algal biomass, consistent with the fact that it is the co-product remaining after the extraction of this protein to produce Spirulysat^®^. Moreover, in Spirugrass^®^, phycocyanin distributed in two peaks of different molecular mass (Figure 3b), the first one in fractions 10–14, between >75 and 60 kDa, and the second one in fractions 14–17 at 35–25 kDa, suggests the presence of different aggregation states of the protein. As reported in the literature, phycocyanin is a very sensitive molecule to structural modifications [24]. A small peak of absorbance at 620 nm is also present in fractions 25–27.

The purity index of phycocyanin, calculated using the A620/A280 ratio, is used to assess the protein purity throughout the purification process, as well as its suitability for various applications [25]. In *L. platensis* extracts, a purity index of 2.07 ± 0.14 was obtained in fraction 12, while a lower value of 0.72 ± 0.13 was obtained for Spirugrass^®^ extracts in fraction 16 (Figure 3c). This index is important, because it determines the market value and potential application of phycocyanin extracted from microalgae. Phycocyanin is considered to be of food, cosmetic, reagent, or analytical grade when the A620/A280 ratio is greater than 0.7, 1.5, 3.9, or 4, respectively [25,26]. The simple purification protocol used in this study, which included soluble protein extraction and SEC, enabling us to obtain a cosmetic-grade purity index for *L. platensis* biomass in fraction 12 and a food-grade purity index for Spirugrass^®^ in fraction 16.

#### 2.1.3. Iron Content and Speciation

*L. platensis* is characterized by high iron levels; however, large variations have been recently reported in commercial samples of *L. platensis* [9]. Therefore, the iron content of *L. platensis* biomass, Spirugrass^®^, and Spirulysat^®^ was measured using AAS. The iron content measured in the algal biomass (374 ± 94 μg/g dry weight) falls within the range of values reported by other authors in commercial samples of *L. platensis*. Principe et al. (2020) [27] reported iron levels from 63 to 1066 µg/g dry weight, Rutar et al. (2022) [28] reported values varying from 370 to 3480 µg/g dry weight, and finally Isani et al. (2022) [9] reported values from 353 to 1459 µg/g dry weight. Interestingly, Spirugrass^®^ retains a consistent iron content (261 ± 15 μg/g dry weight) equivalent to 74% of that present in the algal biomass, suggesting a potential use in animal supplementation. For example, the European Pet Food Industry Federation (FEDIAF) suggests that the minimum iron requirement for adult dogs is 9 mg per 1000 kcal of metabolizable energy [29], and Spirugrass^®^ could be used to supplement iron in diets deficient of this essential element. Finally, Spirulysat^®^ had an iron concentration of 5.87 ± 3.37 µg/mL.

**Figure 4:**
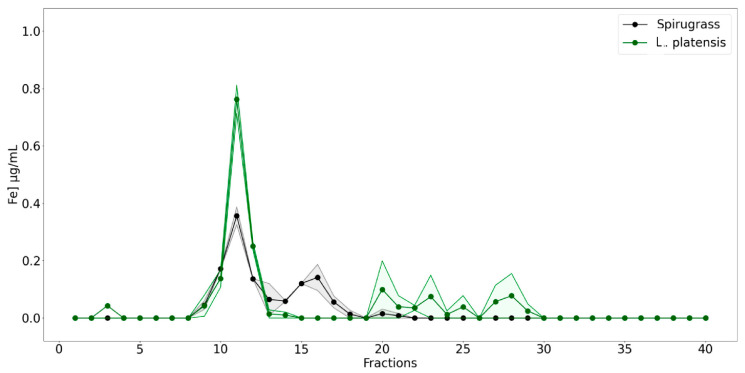
Iron concentrations in SEC fractions obtained from extracts of *L. platensis* and Spirugrass^®^. The shaded area surrounding the chromatograms indicates the variability between replicates.

Iron speciation was studied by combining a chromatographic fractionation of proteins using SEC associated with sensitive metal detection in fractions using AAS, a hyphenated approach which is commonly used in metallomic studies. Iron content was therefore measured in all the fractions obtained from the SEC of *L. platensis* and Spirugrass^®^ extracts (Figure 4). In both *L. platensis* and Spirugrass^®^ extracts, a major peak of iron was detected between fractions 10 and 13, indicating that the element is bound to high-molecular-mass proteins between >75 kDa and 60 kDa. These profiles are consistent with those reported by Isani et al. (2022a) [9] in extracts of *L. platensis*: the major peaks of iron overlap with the peaks of phycocyanin (Figure 3b), confirming the hypothesis that this protein can bind iron. In addition to the major peak in fractions 10–13, a second peak of iron overlapping the second peak of phycocyanin (Figure 3b) was detected between fractions 14–17 in Spirugrass^®^, suggesting that iron was also bound to intermediate-molecular-mass ligands, probably phycocyanin, at lower aggregation state.

### 2.2. Effect of HPP on Spirugrass^®^ Proteins

**Figure 5:**
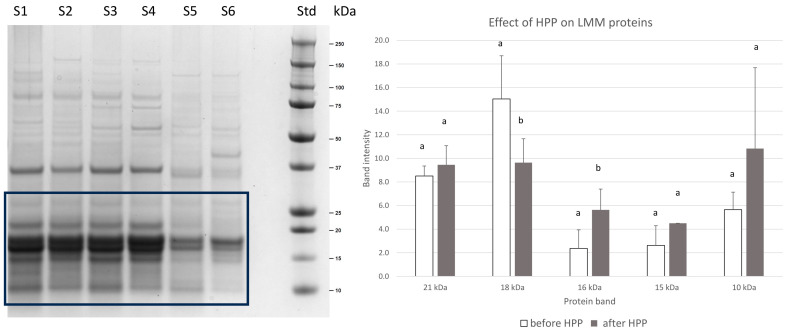
(**Left**): representative SDS-PAGE gel (4–12%, Comassie staining). Spirugrass^®^ extracts after (S1, batch B1; S3, batch B2; S5, batch B4) and before (S2, batch B1; S4, batch B2; S6, batch B4) HPP. Std: molecular mass marker. The black rectangle encompasses the low-molecular-mass (LMM) protein bands which have been quantified. (**Right**): the effect of HPP on low-molecular-mass protein bands (LMM) (between 25 and 10 kDa) is reported. Different letters for each protein band indicate a significant difference (*p* < 0.05) due to HPP treatment.

The second phase of the study evaluated the effect of high-pressure processing (HPP) on the Spirugrass^®^ proteome. The application of HPP at 600 MPa for 5 min, a standard protocol applied in food industry, was explored as a processing treatment to reduce the total microbial charge, improve the stability of this co-product along the time, and make it compliant with the food and feed specification requirements [10]. To the authors’ knowledge, this is the first study investigating algal protein modification using HPP. Electrophoresis was used to separate the proteins in extracts of Spirugrass^®^ and to evaluate the effect of HPP. A representative SDS-PAGE gel of protein extracts from three different batches of Spirugrass^®^ before and after HPP is shown in Figure 5. SDS-PAGE confirmed that there was no severe protein degradation after HPP. However, after the treatment, differences in the protein profiles were detected (Figure 5). In particular, a significant decrease (*p* = 0.039) in the intensity of the 18 kDa protein band, corresponding to phycocyanin, was recorded, suggesting partial protein degradation. Moreover, a significant increase (*p* = 0.01) in the intensity of the 16 kDa protein band was recorded. The protein bands at 15 and 10 kDa also showed an increase; however, this increase was not significant (*p* = 0.06 and *p* = 0.07).

**Figure 6:**
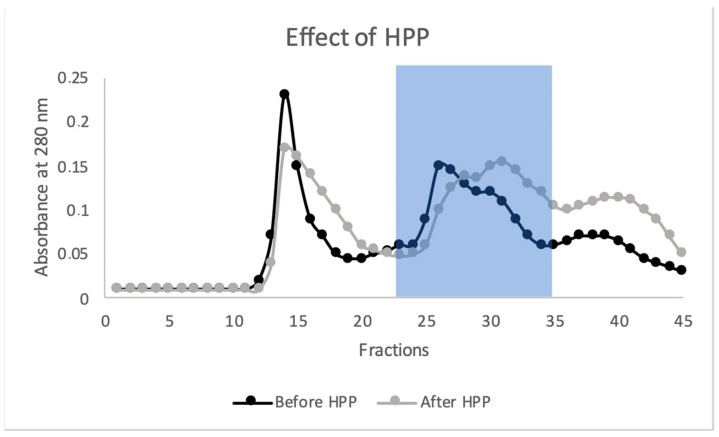
HPLC chromatograms of batch 4 of Spirugrass^®^ before HPP and after HPP. Proteins were detected at 280 nm. The fractions containing phycocyanin are indicated within the blue background rectangle (fractions 23–35).

To further investigate the effects of the HPP treatment on the protein profile under native conditions, the extracts were fractionated by SEC in HPLC. Representative chromatograms of Spirugrass^®^ extracts, obtained by reading the absorbance at 280, before and after the treatment, are shown in Figure 6. Before HPP, the chromatogram showed four peaks, while after HPP, the first peak, containing high-molecular-mass proteins (≥160 kDa), became lower and broader and the peaks containing intermediate and low-molecular-mass proteins, including phycocyanin, shifted towards low-molecular-mass fractions, mirroring the profiles obtained after SDS-PAGE. It has been reported that phycocyanin is very sensitive to structural modifications, due to temperature, pH, solvent, and extraction techniques such as high pressure. A pressure of 600 MPa resulted in 50% of color loss and seemed likely to be related to modifications of phycocyanin structure [24]. Furthermore, the hypothesis of structural modification or partial degradation of high-molecular-mass proteins may be confirmed by the increase in the fourth peak of the chromatogram, which contains low-molecular-mass peptides.

Protein hydrolysis per se is not a negative factor, as publications have reported that certain peptides present in hydrolysates obtained from microalgae may have several interesting activities from a nutraceutical or pharmacological point of view [30]. For example, Aiello et al. (2019) [22] have reported that hydrolysates of spirulina obtained with pepsin and trypsin produced peptides with interesting in vitro antidiabetic and antihypertensive properties, while Lee et al. (2022) [31] have reported that hydrolysates obtained from *L. platensis* inhibit muscle atrophy induced by dexamethasone in C2C12 myotubes and promote muscle regeneration. Finally, Htoo et al. (2024) [20] showed that protein hydrolysates from spirulina could serve as a potential source of bioactive peptides to enhance immunostimulant activity.

### 2.3. Possible Application of Spirugrass^®^ as a Nutritional Supplement for Honeybee Health

A recent review of the potential effects of algae as nutritional supplement in honeybees reported beneficial effects at the colony level on fertility, brood rearing, colony population size, and honey and wax production [6]. These data have encouraged us to expand the research into other interesting biomarkers. Hemolymph proteins, especially apolipophorins, vitellogenin, and hexamerin 70a, are considered to be good biomarkers of honeybee health and nutritional status [32,33]. Therefore, we have used a panel of these proteins previously tested under field conditions [33] as a molecular tool to assess the safety and the nutritional effect of Spirugrass^®^ in comparison with a commercial nutritional supplement commonly used by beekeepers to integrate the honeybee diet during periods of stress or before the winter.

No mortality or other adverse effects, such as diarrhea, were observed, and honeybees accepted and consumed the nutritional supplement containing Spirugrass^®^. This is in agreement with the observation of Ricigliano et al. [34], who found a negligible mortality rate of 3–5% in bees fed spirulina for 10 days compared to pollen- and sugar-fed controls. However, other researchers reported a higher mortality in honeybees fed pollen or pollen substitute when spirulina was added to the diet at different concentrations [35].

The concentration of hemolymph total proteins, a biomarker of health and nutritional status, was within the range of those reported by Rudelli et al. (2024) [33] (17.0–40.5 mg/mL), Isani et al. (2023) [36] (13.3–44.5 mg/mL), and Kunc et al. (2019) [37] (12–42 mg/mL) for healthy honeybees. No significant differences were found between honeybees supplemented with Spirugrass^®^ and those of the negative or positive control groups (Table 1).

**Figure 7:**
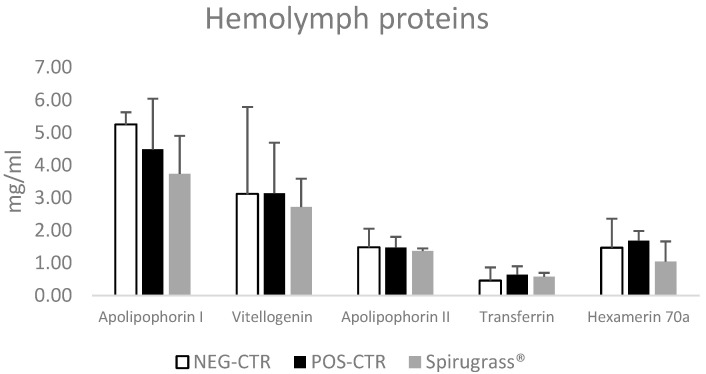
Concentrations of apolipophorin I and II, vitellogenin, transferrin, and hexamerin 70a in the hemolymph of honeybees fed, respectively, with sugar syrup (Neg-CTR), Promotor L apis, (POS-CTR), and Spirugrass^®^. Data are expressed as mg/mL and reported as mean ± SD (n = 3).

The most abundant hemolymph proteins, namely apolipophorin I, vitellogenin, apolipophorin II, transferrin, and hexamerin 70a, could be separated and quantified in all the samples analyzed; the data are reported in Figure 7. This panel of proteins can be used to assess the nutritional and health status of honeybees, as previously reported by Rudelli et al. [37]. The profiles of hemolymph proteins obtained after SDS-PAGE were consistent with those reported in healthy honeybees by other authors [36,38,39]. No significant differences in these proteins were found between honeybees fed with the commercial supplement or sugar syrup and those supplemented with Spirugrass^®^, suggesting that the supplement has no negative effects on the physiological functions and metabolism of honeybees.

The effects of spirulina supplementation in honeybees were studied by Ricigliano et al. (2020) [34], who reported that vitellogenin mRNA levels in spirulina-fed bees matched that of pollen-fed bees and were higher than those measured in control sugar-syrup-fed bees. However, vitellogenin concentrations obtained in this study (3.11 ± 2.67 mg/mL for the NEG-CTR, 3.14 ± 1.55 mg/mL for the POS-CTR, and 2.09 ± 2.29 mg/mL for honeybees fed with Spirugrass^®^) are lower than those reported by Rudelli et al. (2024) [37] (9.54–15.8 mg/mL) and Isani et al. (2023) [36] (5.70–12.5 mg/mL). In both studies, honeybees were collected in July; therefore, the sampling period is the same as that of this study and should not affect protein concentration. Other factors may have influenced the concentration of vitellogenin, the most important being the age of the honeybees, which were only 4–5 days old in this study. It has been reported that vitellogenin concentration varies according to the role that honeybees play in the colony (nurses or foragers) [39] and increases in newly emerged bees from the birth reaching a maximum at day 12 [40]. In addition, the different experimental settings of this study, which was conducted in cages rather than in the field at the colony level, could have influenced protein concentration.

Finally, the high iron content in Spirugrass^®^ could be advantageous in this context, because honeybees require iron for orientation [41], in addition to other essential physiological and metabolic functions carried out in cells by this element. To the authors’ knowledge, no information is available regarding the nutritional iron requirements of honeybees. However, in previous research, we have observed an inverse relationship between *Varroa* infestation and iron content in August [37], and Rodríguez-García et al. (2021) [42] have reported that *Nosema ceranae* infection causes iron deficiency in honeybees. By sustaining iron status, Spirugrass^®^ could help mitigate some of the adverse consequences of *N. ceranae* infection, such as energetic stress and reduced longevity [43], thus supporting colony resilience. Indeed, a nutritional supplement such as Spirugrass^®^, might help to maintain an appropriate intake of this essential trace element. This could be particularly important at the end of summer, when honeybees prepare for the wintering phase, which represents a critical period for the colonies.

## 3. Material and Methods

### 3.1. Characterization of Spirugrass^®^ and Comparison with the Algal Biomass

In the first part of this study, two different samples of *L. platensis* (total dried biomass) and three batches (SPI80.21274-1, SPI81.22063, SPG21288-1) of Spirugrass^®^, the co-product obtained from the algal biomass after the extraction of bioactive molecules, were analyzed. Three samples of Spirulysat^®^, a purified aqueous extract of *L. platensis* containing phycocyanin, were also included for comparison purposes. All of these samples were provided by Algosource (Le Frostidié 44410, Assérac, France). *L. platensis* biomass and Spirugrass^®^ were freeze-dried until reaching a moisture content of 5–6%. The dried powders were stored in the dark at room temperature until use. From each sample, soluble proteins were extracted, fractionated using SDS-PAGE electrophoresis and size exclusion chromatography, and finally, the iron content and speciation were assessed.

### 3.2. Soluble Protein Extraction from Algal Samples and Iron Quantification

The extraction of soluble proteins was performed starting from *L. platensis* dried biomass and Spirugrass^®^. The extraction of the soluble fraction was carried out following the procedure reported by [9]. One-hundred milligrams of the dried biomass of Spirugrass^®^ or *L. platensis* were diluted 1:30 *w/v* in Tris-HCl 20 mM, pH 8.0 and 5 mM 2-mercaptoethanol. The samples were vortexed 3 times for 10 s and then incubated for 3 h at 4 °C. Afterwards, samples were homogenized with Ultra-turrax T25 (Janke & Kunkel; IKA^®^-Labortechnik) at 20,500 rpm 3 times for 10 s and then centrifuged at 18,000× *g* for 40 min at 4 °C, obtaining the separation between supernatant and pellet. Samples were stored at −20 °C until analysis. Each extraction was performed in triplicate.

Protein concentration in extracts was determined by the Lowry method using BSA as a standard (Sigma-Aldrich, St. Louis, MO, USA).

#### 3.2.1. SDS-PAGE of Protein Extracts

The proteins of Spirulysat^®^ and of extracts of Spirugrass^®^ and *L. platensis* were fractionated on 4–12% polyacrylamide precast gels (NuPage, Thermo Fisher Scientific, Waltham, MA, USA).

Samples were added with lithium dodecyl sulfate (LDS; Thermo Fisher Scientific, Waltham, MA, USA) and a reducing agent (Invitrogen, Thermo Fisher Scientific, Waltham, MA, USA) and denatured at 70 °C for 10 min. SDS-PAGE was carried out in Novex Mini-Cell (Invitrogen, Thermo Fisher Scientific, Waltham, MA, USA) and the running buffer used was 2-morpholinoethanesulfonic acid (MES) (Nupage, Thermo Fisher Scientific, Waltham, MA, USA). Depending on the dilution, 10 or 5 micrograms of protein were loaded for each sample. Each gel was also loaded with standard proteins of known molecular mass (Precision Plus ProteinTM Dual Color Standard, Bio-Rad, Hercules, CA, USA). The electrophoresis system was connected to a power supply (Power Pack Basic Bio-Rad, Hercules, CA, USA) at a constant voltage of 200 V. The gels were stained with Quick Coomassie Stain (Protein Ark, Sheffield, UK). After destaining, each gel was digitalized using ChemiDocMP (Bio-Rad, Hercules, CA, USA), and the pherograms were obtained using the ImageLab 5.2.1 software (Bio-Rad, Hercules, CA, USA). Band densitometry was performed for quantitative comparison between samples before and after HPP treatment. To determine the band intensity, the software determines the volume of each protein band through the analysis of the pixel values in the digital image—this refers to the volume of the sum of all the pixel intensities within the band boundaries.

#### 3.2.2. Size Exclusion Chromatography (SEC)

A volume of 0.8 mL of supernatant was applied to a Sephadex G-75 column (0.9 × 90 cm). The column was calibrated using a commercially available kit (GF70-1KT, Sigma-Aldrich, St Louis, MO, USA) and the buffer used was Tris-HCl 20 mM, pH 8.0 with 5 mM 2-mercaptoethanol. Fractions of 1.5 mL were collected.

The total proteins and phycocyanin were analyzed by measuring the absorbance at 280 nm and 620 nm, respectively. The phycocyanin purity index was calculated as the ratio of phycocyanin absorbance at 620 nm to the total soluble protein absorbance at 280 nm [44]. Iron concentration was measured by direct aspiration of the solutions into an atomic absorption spectrophotometer (AAnalyst 100, Perkin Elmer, Waltham, MA, USA), as reported in Section 3.2.3. As for proteomic analysis, SDS-PAGE of fractions was performed following the protocol reported in Section 3.2.1.

#### 3.2.3. Iron Analysis Using Atomic Absorption Spectrometry

Atomic absorption spectrometry was used to detect the iron content in the samples of *L. platensis*, Spirugrass^®^ and Spirulysat^®^. All the reagents were from Merck (Darmstadt, Germany) and the acids were of Suprapur grade. Two hundred milligrams of the sample were placed in Teflon tubes and were digested with 2 mL 65% HNO_3_ and 0.5 mL 30% H_2_O_2_ in a microwave oven for 5 min at 250 W, 5 min at 400 W, 5 min at 500 W, and 1 min at 600 W. The cooled samples were transferred into 10 mL polyethylene volumetric flasks and then analyzed using a flame atomic spectrophotometer (AAnalyst 100, Perkin Elmer, Waltham, MA, USA). Fractions obtained from SEC and Spirulysat^®^ were directly analyzed without any further treatment. The limit of detection for iron was 0.04 µg/mL. Iron concentration (in SEC fractions and in Spirulysat^®^) or content (in Spirugrass^®^ and algal biomass) was reported as µg/mL or µg/g dry weight (dw), depending on the sample analyzed.

### 3.3. HPP Treatment

HPP treatments were carried out on three independent batches of Spirugrass^®^ with Wave 6000/120 industrial equipment (Hiperbaric S.A., Burgos, Spain) at 600 MPa for 5 min. Samples were packed and sealed in multilayer aluminum/plastic bags and submitted to an HPP at ambient temperature (20 °C). The HPP working parameters (600 MPa for 5 min at 20°) were chosen according to literature examples in foods [45,46,47]. During the HPP treatment, pressure increased linearly in 3 min up to 600 MPa, maintained for 5 min and then suddenly lowered to ambient pressure. The increase in sample temperature during the HPP treatment can be estimated as approximately 3 °C for every 100 MPa of applied pressure, while the initial and final temperatures of the sample were identical due to the adiabatic conditions. HPP-treated Spirugrass^®^ was freeze-dried until reaching a moisture content of 5–6%. The dried powders were stored in the dark at room temperature until use.

### 3.4. Effect of HPP on Spirugrass^®^ Proteins

To evaluate the effect of HPP on Spirugrass^®^ proteins, we opted to use HPLC, which allows for greater resolution and separation efficiency, leaving proteins in their native state. One milliliter of the supernatant previously obtained from the extraction of soluble proteins (see Section 3.2) was injected into an HPLC system. The mobile phase for HPLC was Tris-HCl 20 mM and NaCl 0.15 M; 37% HCl was added drop by drop until a pH 8.0 was obtained and then filtered with 0.2 μm 47mm GHP membrane (Pall Corporation; 600 South Wagner Road; Ann Arbor, Michigan 48103-9019). HiPrep 16/60 Sephacryl S-300 High Resolution column (GE Healthcare Bio-Sciences, Uppsala, Sweden) was used as stationary phase. The column was washed with 60 mL of bi-distilled water at 500 μL/min for 2 h, then with buffer at 500 μL/min overnight. Chromatography was carried out with the following parameters: AT = 1024, CS = 0.1, Flow rate 1.000 (1 mL/min). The system was plugged in with a 280 nm detector. Fifty fractions of 1.5 mL were collected and stored at −20 °C.

### 3.5. Honeybees and Experimental Feeding Trial

In July 2023, brood frames were collected from a hive of healthy Italian honeybees (*Apis mellifera ligustica*) located in the district of Bologna and incubated in the laboratory at 32 ± 2 °C and 60% RH until the honeybees emerged. Then, 9 groups of 30 newly emerged honeybees were collected and placed in 9 transparent and ventilated plastic boxes (12 cm × 8 cm × 6 cm). Three groups were used as negative control (fed with sugar syrup only) [NEG-CTR] and three groups were used as positive control (fed with Promotor L Apis, a commercial supplement) [POS-CTR], while three groups were fed with Spirugrass^®^. Briefly, 1 g of Spirugrass^®^ was suspended in 10 mL of a 2:1 (sugar/water *w*/*w*) sugar syrup solution. Half a milliliter of the resulting suspension was administered daily to each cage using a gravity feeder for four days. Cages were maintained at an average temperature of 29 ± 2 °C and 60% relative humidity (RH) throughout the feeding trial. From each cage, 10 honeybees were randomly selected and anesthetized in ice; 1–2 µL of transparent uncontaminated hemolymph was collected from each bee, as described by Cabbri et al. (2018) [39]. Samples were stored at −80°.

#### 3.5.1. Hemolymph Total Protein Determination

Total protein (TP) concentration was determined in hemolymph using the Bradford method (Bradford Reagent, Sigma-Aldrich, St. Louis, MO, USA). Bovine serum albumin (Sigma-Aldrich, St. Louis, MO, USA) served as the standard for the calibration curve. Absorbance was measured with a plate reader (VarioskanTM Lux, Thermo Fisher Scientific, Waltham, MA, USA).

#### 3.5.2. SDS-PAGE of Proteins in Honeybee Hemolymph

Hemolymph proteins were separated and quantified by 1D-SDS-PAGE electrophoresis. Samples were diluted to obtain 3 µg of total protein and were loaded onto 4–12% Bis-Tris polyacrylamide gels (NuPage/Thermo Fisher Scientific, Waltham, MA, USA); electrophoresis was performed at a constant voltage of 200 V for 40 min as described in Isani et al., 2023 [36]. Each gel contained standard proteins of known molecular mass (SeeBlue™ Plus2 Pre-stained Protein Standard, Thermo Fisher Scientific, Waltham, MA, USA). Gels were stained with Coomassie G250. After staining, each gel was digitized using ChemiDocMP (Bio-Rad, Hercules, CA, USA), and pherograms were obtained with ImageLab 5.2.1 software (BioRad, Hercules, CA, USA). The quantification of protein bands was performed as described in Isani et al. (2023) [36], using an internal standard of quantity (1 µg of protein) obtained by diluting 1:5 *v:v*, a commercial solution of LDH (5 µg/µL) (Sigma-Aldrich/Merck, Darmstadt, Germany) containing 5 mg of protein/mL.

### 3.6. Statistical Analysis

The chromatograms in Figure 3 and Figure 4 were obtained using Matplotlib (https://matplotlib.org/), a Python library dedicated to high-quality plotting, and Numpy (https://numpy.org/), a Python library for statistical and numerical analysis.

As for hemolymph proteins, the distribution and homoscedasticity of variables in each group were assessed through Shapiro–Wilk and Levene test, respectively [48]. When distribution was not normal, Kruskal–Wallis and Dunn post hoc test for multiple comparison were applied. Conversely, when distribution was normal and data had equality of variances, one-way ANOVA with Tukey HSD post hoc test were applied. Statistical analyses were performed using R 4.3.2 (R foundation for statistical computing; Vienna, Austria; https://www.R-project.org/ accessed on 28 October 2024). A *p*-value < 0.05 was considered statistically significant.

## 4. Conclusions

SDS-PAGE and SEC are useful techniques for checking the quality and integrity of the Spirugrass^®^ proteome, which still contains a significant amount of soluble proteins, including phycocyanin, compared to algal biomass. Investigating iron speciation using SEC and AAS revealed that, in both *L. platensis* and Spirugrass^®^, iron is primarily bound to high-molecular-mass proteins, including phycocyanin, thus increasing its bioavailability. Applying HPP to Spirugrass^®^ as a stabilization process caused changes to the protein profile, resulting in the production of small-molecular-mass proteins or peptides. The presence of these biomolecules should be investigated in future studies.

The presence of consistent levels of soluble proteins, as well as protein-bound iron, suggests that Spirugrass^®^ may be considered a good candidate as a feed supplement. Preliminary data obtained with honeybees are encouraging, as no mortality or adverse effects were observed, and the hemolymph protein profile was not significantly different from either the negative or the positive control. However, Spirugrass^®^ supplementation did not yield significant beneficial effects compared with sugar feeding. Therefore, sugar syrup remains the more economically viable option for beekeepers. Nevertheless, Spirugrass^®^ may represent a sustainable opportunity for the valorization of this co-product within a circular economy approach and further studies on the impact on the nutritional response in apiary at the colony level are required.

Food production will pose a significant challenge in the future, and it is crucial to find sustainable alternatives, such as the reuse of waste or by-products. Further research is needed to understand how HPP affects protein structures and to optimize conditions for preserving the integrity of valuable components of Spirugrass^®^.

## Figures and Tables

**Table 1:** Concentration of hemolymph total proteins in honeybees fed, respectively, with sugar syrup (NEG-CTR), Promotor L apis, (POS-CTR), and Spirugrass^®^. The data are expressed in mg/mL and reported as the mean ± SD (n = 3).

Experimental Conditions	Total Proteins (mg/mL)
NEG-CTR	16.80 ± 4.41
POS-CTR	24.52 ± 10.15
Spirugrass^®^	18.94 ± 9.25

## Data Availability

The original contributions presented in this study are included in the Supplementary Material. Further inquiries can be directed to the corresponding authors.

## Supplementary Materials

The following supporting information can be downloaded at: https://www.mdpi.com/article/10.3390/md23110443/s1, Dataset: Dataset.
